# Abnormalities in one-carbon metabolism in young patients with psychosis

**DOI:** 10.3389/fpsyt.2023.1128890

**Published:** 2023-02-01

**Authors:** Ariel Frajerman, Marie Urban, Fabrice Rivollier, Marion Plaze, Boris Chaumette, Marie-Odile Krebs, Linda Scoriels

**Affiliations:** ^1^Université de Paris, INSERM U1266, Institute of Psychiatry and Neuroscience of Paris (IPNP), CNRS GDR3557, Paris, France; ^2^MOODS Team, INSERM, CESP, Université Paris-Saclay, Faculté de Médecine Paris-Saclay, Le Kremlin-Bicêtre, France; ^3^Service Hospitalo-Universitaire de Psychiatrie de Bicêtre, Mood Center Paris Saclay, Assistance Publique-Hôpitaux de Paris, Hôpitaux Universitaires Paris-Saclay, Hôpital de Bicêtre, Le Kremlin-Bicêtre, France; ^4^Groupe Hospitalier Universitaire (GHU) Paris Psychiatrie et Neurosciences, Paris, France; ^5^Department of Psychiatry, McGill University, Montreal, QC, Canada

**Keywords:** folic acid, personalised medicine, early intervention, early psychosis, one-carbon metabolism

## Abstract

**Introduction:**

Folates, the main actors in one-carbon (C1) metabolism, are involved in synthesising monoamines and maintaining genomic stability. Previous studies support the association between C1 metabolism and schizophrenia. The main purpose of this study was to assess the prevalence of plasma folate, and/or vitamin B12 deficiencies and hyperhomocysteinemia in young patients with psychotic disorders.

**Methods:**

We included young inpatients (15–30 years old) with psychosis between 2014 and 2017 from Sainte-Anne Hospital in Paris. Plasma folate, vitamin B12 deficiency and homocysteinemia dosages were done at admission. Clinical data were extracted retrospectively, and patients diagnosed with a first-episode psychosis (FEP), schizophrenia, schizoaffective disorder, or persistent delusional disorder were retained for the analysis.

**Results:**

Among the 334 inpatients, 188 (56%) had C1 dosages available (135 males; 53 females). From the 188 patients, 32% had a C1 abnormality. This abnormality reached 38% of FEP patients. The most frequent abnormality was folate deficiency: 21% of all patients and 27% of FEP. Lower levels of folates were found in males compared to females (*p* = 0.02) and were correlated with more severe disorder, as assessed by Clinical Global Impression – Severity (CGI-S; *p* = 0.009). Antipsychotic dosage was positively associated with B12 levels (*p* = 0.013) and negatively with homocysteinemia (*p* = 0.034).

**Conclusion:**

One-carbon metabolism anomalies in young patients with psychotic disorders are highly prevalent, reaching almost half of the patients with FEP. Potential protective effects from females and antipsychotics have emerged. These results spotlight the need for new therapeutic prospects, such as folate supplementation, to achieve personalised medical approaches to the early stages of psychotic disorders.

## Introduction

Schizophrenia is a mental illness that affects 1% of the world’s population, i.e., approximately 600,000 people in France. This pathology results from a combination of genetic and environmental factors (1). According to a gene x environment interaction model (2), genetically vulnerable individuals are at greater risk of developing schizophrenia after being exposed to certain environmental factors. Identifying risk factors, especially the ones that can be altered, is the objective of numerous scientific studies. The one-carbon cycle (C1) is a set of biochemical reactions centred on folate metabolism, which is at the interface between different etiopathogenic factors of schizophrenia, such as abnormal transmission of monoamines, epigenetic dysregulation, oxidative stress, and maternal hyperhomocysteinemia (1).

Folates, the main players in C1 metabolism, are essential for monoamine synthesis and genome stability, as they regulate DNA synthesis, repair, and methylation. Dihydrofolate, the form of folate naturally present in our diet (3), is gradually converted into L-methylfolate in the digestive tract by the methylenetetrahydrofolate reductase (MTHFR), one of the key enzymes of this metabolism (4), which gene is associated with schizophrenia (5). L-methylfolate is the main active form of folate available in the body, it crosses the blood-brain barrier and is converted into tetrahydrofolate. Tetrahydrofolate derivatives are involved in amino acid and nucleotide metabolism reactions, participating in thymidine, adenine, and guanine synthesis. The folate cycle also involves the dihydrofolate reductase that allows the rescue of tetrahydrobiopterin (BH4), which acts as a cofactor for enzymes producing dopamine, serotonin, and nitric oxide (6). In the methionine cycle, L-methylfolate remethylates, thanks to vitamin B12 cofactor, homocysteine into methionine. Methionine is then transformed into S-adenosylmethionine (SAM), the principal methyl donor in the body, which controls gene transcription and protein expression through its ability to methylate DNA CpG islands in the promoter regions of genes (epigenetic phenomenon) (7).

Folate availability is regulated by several factors, such as the nutritional intake of folate, its absorption through the gut, its transport within cells and the enzymatic conversion to active forms (8). Factors such as age, pregnancy, malnutrition or the use of drugs that reduce folate level (for instance antiepileptics) (9) are associated with vitamin deficiencies and dysfunctions in the C1 pathways. The quality of the diet is also of crucial importance. Indeed, Marchetta et al. estimated that a 10% increase in natural dietary folate intake would increase intra-erythrocyte folate concentration by 6% and plasma folate concentration by 7% (10).

Two meta-analyses described lower mean plasma folate concentrations in patients with schizophrenia (1,463 and 1,773 patients respectively in each study) compared to healthy controls (1,276 and 1,930 controls respectively in each study) (11, 12). An association has also been found between increased plasma homocysteine concentrations and schizophrenia (13). An increase of 5 μmol/L in homocysteinemia appears to correspond to a 70% (OR 1.7) inflation of the risk of schizophrenia (14). A Mendelian randomisation study also showed that elevated plasma homocysteine levels could increase the risk of schizophrenia (15). Folate deficiency and hyperhomocysteinemia appears to be more specifically associated with negative symptoms and cognitive alterations in this population (16).

Nevertheless, the relationships between folate and homocysteine levels in psychosis onset still need to be clarified, considering certain methodological pitfalls: absence of well-defined international standards, different analysis techniques depending on the laboratory, non-matching of cases and controls on age or sex, failure to take into account dietary habits, etc. (17).

In patients with first-episode psychosis (FEP), several studies have found lower mean plasma and intra-erythrocyte folate levels and higher homocysteinemia compared to healthy controls, including in treatment-naive patients (18–20). In addition, a metabolomics study found that molecules from the C1 were under-expressed in the serum of FEP patients, as compared to their first-degree relatives and even more when compared to healthy controls (21). Another study also showed that a family history of first or second-degree schizophrenia or cannabis use was associated with significantly higher homocysteinemia in FEP patients (22).

The main objective of this study was to assess the prevalence of deficiencies in several molecules involved in the C1 cycle [i.e., folate (B9), vitamin B12, and homocysteine] in a cohort of young patients with psychotic disorders in order to disentangle the relationship of these molecules between them and across diagnostics related to early psychosis. Our hypothesis was that C1 abnormalities would be substantially higher in patients with schizophrenia as compared to FEP. We also conjectured that the changes in all the three molecules (i.e., folate, vitamin B12, and homocysteine) would be interdependent, considering they belong to the same pathways. Secondary objectives were to investigate the potential relationship between folate levels and specific socio-demographic and clinical characteristics, such as sex, age, and treatment.

## Materials and methods

### Population

This single-centre retrospective study was conducted in the University Hospital Service (SHU) at Sainte-Anne Hospital in Paris. We enrolled all inpatients, aged between 15 and 30 years, between 2014 and 2017, with a plasma folate assay after admission and a diagnosis of psychotic disorder (FEP, schizophrenia, schizoaffective disorder or persistent delusional disorder, according to the DSM 5 criteria). The 15–30 years age group was targeted because of our specific interest in early psychosis and to minimise age-related folate deficiency or homocysteine elevations. Patients who were treatment naïve or new to care were also less likely to have iatrogenic folate deficiency or supplementation.

### Data collection

An initial selection was made based on age and primary diagnosis from all patients hospitalised in the SHU between January 2014 and September 2017. Patients hospitalisation reports were then extracted to verify whether the discharge diagnosis corresponded to the inclusion criteria.

Biological results were transmitted by the hospital laboratory or obtained via the computerised software DxCare©. These included plasma levels of folate (B9), B12, and homocysteine. Whenever biological results were available, the following data were also collected: age on admission, sex, antipsychotic treatment on admission (chlorpromazine equivalent), use of an anticonvulsant treatment, primary diagnosis, addictive comorbidity (alcohol or cannabis), existence of an eating disorder, initial severity of the disease assessed by the Clinical Global Impression – Severity (CGI-S) and prescription of vitamin supplementation during hospitalisation. Finally, these data were anonymised and entered into a spreadsheet as continuous quantitative variables or binary qualitative variables.

### Biological measures

The primary endpoint was the prevalence of folate deficiency, vitamin B12 deficiency, hyperhomocysteinemia, or one or more of these three conditions in this patient cohort.

Vitamin deficiency and hyperhomocysteinemia were defined by plasma concentrations strictly below or above the standards set by the hospital laboratory. These corresponded to plasma levels below 7 nmol/L for folate and 150 pmol/L for vitamin B12, and above 15.0 μmol/L for homocysteine. All dosages were performed in the same laboratory.

### Statistical analysis

Kruskal–Wallis and Mann–Whitney U were performed to analyse differences between three and two groups respectively. Where appropriate, differences in proportion between diagnostic groups was performed using χ^2^. Spearman’s correlation test was used to test for statistical correlation between folate (vitamin B9) levels and other quantitative variables (age, homocysteinemia, and dosage of antipsychotic medication). A binary logistic regression performed the association between folate level or homocysteinemia and some binary categorical variables (sex and anticonvulsant treatment). The first order risk α was set at 0.05 on the IBM SPSS Statistics V20 software.

## Results

### Population

A total of 334 patients aged 15–30 years with psychotic disorders were admitted to the SHU between January 2014 and September 2017. Of these, 188 (56%) patients had a folate assay and were included in the study. This population consisted of 135 males (72%) and 53 females (28%) who were about 23 years old, took an average of 183 mg of chlorpromazine equivalents and 7.4% were also taking anticonvulsants (Table 1). Among patients, 24% had a diagnosis of FEP, 62% had a diagnosis of schizophrenia, 11% had a diagnosis of schizoaffective disorder and 3% had other diagnoses. Due to overlapping diagnostic criteria, 21 out of 45 patients with FEP (47%) also had a diagnosis of schizophrenia (progression of the disorder for more than 6 months and less than 2 years) but were maintained in the FEP group for the analyses. The three diagnostic groups (i.e., FEP, schizophrenia, and schizoaffective disorder) differed in their dosage of antipsychotic and anticonvulsive medication, which was significantly higher for patients with schizophrenia and schizoaffective disorders compared to FEP (Table 1). The three groups also differed in the initial severity of the disease, as assessed by the CGI-S (*p* = 0.03), and FEP patients also consumed significantly more cannabis compared to patients with schizophrenia and schizoaffective disorders (*p* = 0.02, Table 1). Women in the study were significantly younger (*p* = 0.03) and had four cases of eating disorders (*p* = 0.005) (Table 2).

**TABLE 1:** Clinical and biological characteristics of the whole sample and according to diagnosis.

	All patients (*n* = 188)	First-episode psychosis (FEP; *n* = 45)	Schizophrenia (*n* = 116)	Schizoaffective disorder (*n* = 21)	*p*-value
Age, mean years (SD)	22.9 (4.2)	23.0 (4.2)	22.6 (4.3)	24.6 (4.0)	0.12
CGI-S^#^, mean score (SD)	5.4 (0.8)	**5.7 (0.7)**	**5.3 (0.8)**	**5.5 (1.0)**	**0.03**
Antipsychotics*, mean mg (SD)	**182 (291)**	**64 (189)**	**227 (319)**	**236 (270)**	**3 × 10^–6^**
Anticonvulsant, *N* (%)	**14 (7.4)**	**0 (0)**	**10 (8.6)**	**4 (19)**	**0.02**
Eating disorder, *N* (%)	4 (2.1)	2 (4.4)	2 (1.7)	0 (0)	0.44
Alcohol use disorder, *N* (%)	12 (6.4)	4 (8.9)	6 (5.2)	2 (9.5)	0.60
Cannabis use, *N* (%)	**56 (30)**	**21 (47)**	**28 (24)**	**6 (29)**	**0.02**
Folate (B9), mean nmol/L (SD)	12.9 (7.3)	11.4 (6.2)	13.5 (8.9)	13.0 (8.8)	0.26
B12, mean pmol/L (SD)	309 (118)	315 (148)	312 (108)	296 (101)	0.83
Homocysteine, mean μmol/L (SD)	*12.6 (7.3)*	*14.4 (9.1)*	*11.9 (6.9)*	*11.9 (4.8)*	*0.09*
Folate (B9) deficiency, *N*/total (%)	39 (21)	12 (27)	20 (17)	4 (19)	0.40
Vitamin B12 deficiency, *N*/total (%)	8 (4.3)	2 (4.4)	5 (4.3)	1 (4.8)	0.99
Hyperhomocysteinemia, *N*/total (%)	29 (15)	8 (18)	14 (12)	4 (19)	0.52
One or more C1 abnormality, *N*/total (%)	60 (32)	17 (38)	31 (27)	8 (36)	0.30

^#^CGI-S: Clinical Global Impression – Severity scale. *Chlorpromazine equivalent. Bold values represent statistically significant result (*p* ≤ 0.05) and italic values represent trend for significance (0.05 ≤ *p* ≤ 0.1).

**TABLE 2:** Clinical and biological characteristics according to sex.

	Men [*n* = 135 (72%)]	Women [*n* = 53 (28%)]	*p*-value
Age, mean years (SD)	**23.4 (4.1)**	**21.9 (4.4)**	**0.03**
CGI-S^#^, mean score (SD)	*5.4 (0.8)*	*5.6 (0.7)*	*0.09*
Antipsychotics*, mean mg (SD)	181 (282)	189 (314)	0.84
Anticonvulsant, *N* (%)	11 (8.1)	3 (5.7)	0.57
First episode psychosis (FEP), *N* (%)	*28* (21)	*17* (32)	*0.07*
Schizophrenia, *N* (%)	88 (65)	28 (53)	0.26
Schizoaffective disorder, *N* (%)	17 (13)	5 (9.4)	0.24
Persistent delusional disorder, *N* (%)	3 (2.2)	2 (3.8)	0.24
Eating disorder, *N* (%)	**0 (0)**	**4 (7.6)**	**0.005**
Alcohol use disorder, *N* (%)	2 (1.5)	1 (1.9)	0.28
Cannabis use, *N* (%)	41 (30)	15 (28)	0.86
Folate (B9), mean nmol/L (SD)	**12.2 (8.2)**	**14.4 (8.2)**	**0.02**
B12, mean pmol/L (SD)	307 (117)	314 (120)	0.84
Homocysteine, mean μmol/L (SD)	**13.2 (8.1)**	**11.0 (4.3)**	**0.05**
Folate (B9) deficiency, *N* (%)	31/135 (23)	8/53 (15)	0.23
Vitamin B12 deficiency, *N* (%)	7/133 (5.2)	1/52 (1.9)	0.32
Hyperhomocysteinemia, *N* (%)	23/117 (20)	6/45 (13)	0.33
One or more C1 abnormality, *N* (%)	47/135 (35)	13/53 (25)	0.18

^#^Clinical Global Impression – Severity scale. *Chlorpromazine equivalent. Bold values represent statistically significant result (*p* ≤ 0.05) and italic values represent trend for significance (0.05 ≤ *p* ≤ 0.1).

### One carbon cycle abnormalities

#### Prevalence

**FIGURE 1:**
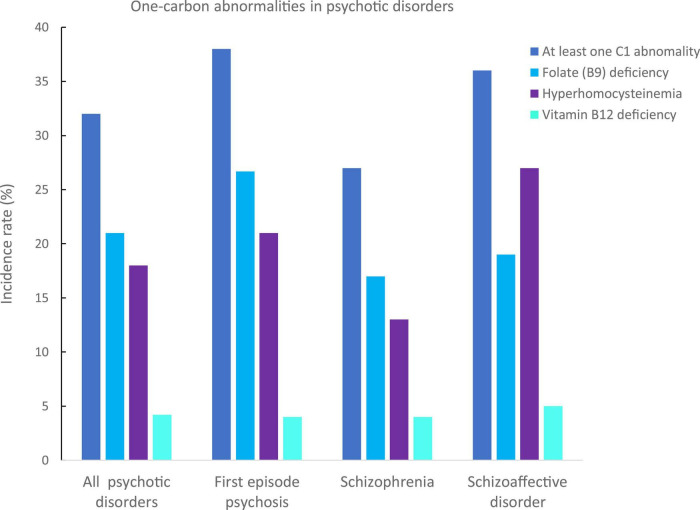
Incidence rates of one-carbon (C1) abnormalities in our psychotic disorders sample. C1 abnormalities have been identified in deficiencies in folates (B9, in light blue), vitamin B12 (in cyan), and hyperhomocysteinemia (in purple). The incidence of at least one C1 in the whole sample was of 32%, 38% for first episode psychosis, 27% for schizophrenia, and 36% for schizoaffective disorders.

In all patients, 32% had at least one C1 abnormality: 38% in FEP patients, 27% in patients with schizophrenia, and 36% in patients with schizoaffective disorders (Figure 1).

The most frequent C1 abnormality was a folate (B9) deficiency with an incidence rate of 21% in all patients: 27% in FEP, 17% in patients with schizophrenia, and 19% in patients with schizoaffective disorder. There were no significant differences between groups (*p* = 0.40, Table 1 and Figure 1).

The second most frequent C1 abnormality was a hyperhomocysteinemia with an incidence rate of 15% in all patients: 18% in FEP, 12% in patients with schizophrenia, and 19% in patients with schizoaffective disorder. There was a trend for difference in homocysteinemia levels between the three groups, which was due to significantly higher levels in FEP compared to schizophrenia patients (*p* = 0.03, Table 1).

No differences were found in vitamin B12 levels between the three groups (*p* > 0.1, Table 1).

**FIGURE 2:**
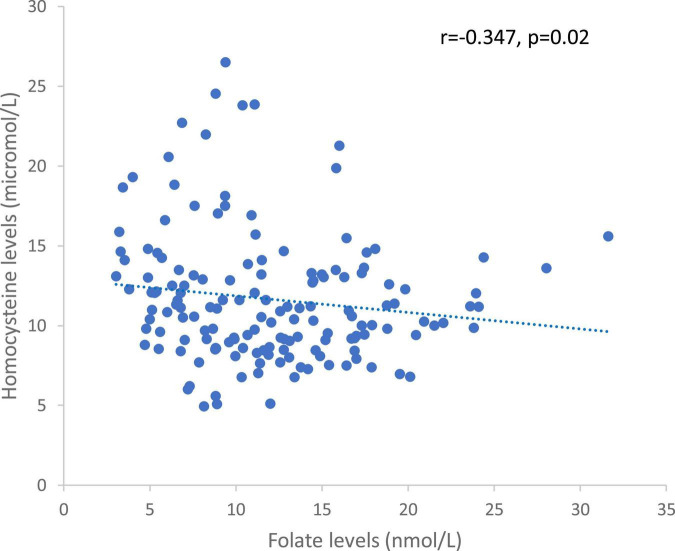
Correlation between homocysteine and folate levels in the whole sample.

Homocysteinemia levels and deficits were negatively correlated with plasma folate levels (rho = −0.347, *p* = 0.02, Figure 2 and rho = −0.620, *p* = 7 × 10^–7^, respectively).

#### Sex and age influence

**FIGURE 3:**
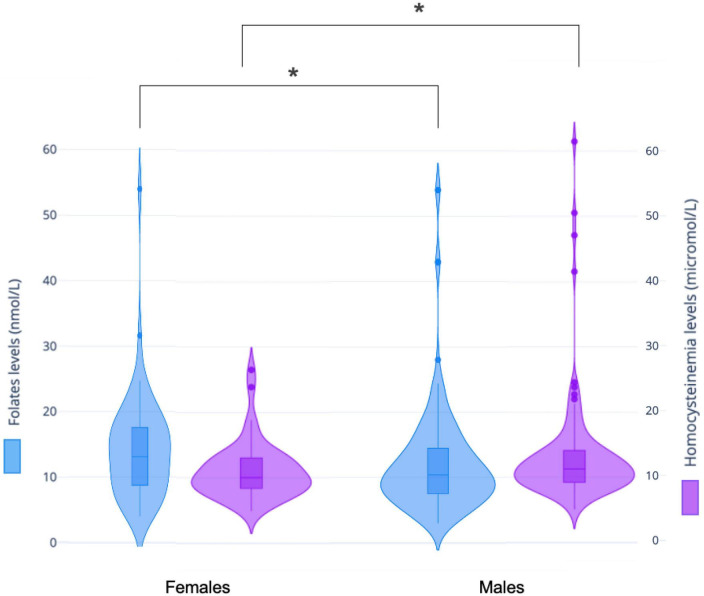
Differences in one-carbon (C1) molecules levels between women and men. Violin and box plots presenting folates and homocysteine levels distribution in men and women. Men presented significantly lower average levels of folate compared to women (12.2 ± 8.2 versus 14.4 ± 8.2 nmol/L, *p* = 0.02, in blue). Men also presented significantly higher average levels of homocysteinemia compared to women (13.2 ± 8.1 versus 11 ± 4.3 μmol/L, *p* = 0.05, in purple). *p < 0.05.

**FIGURE 4:**
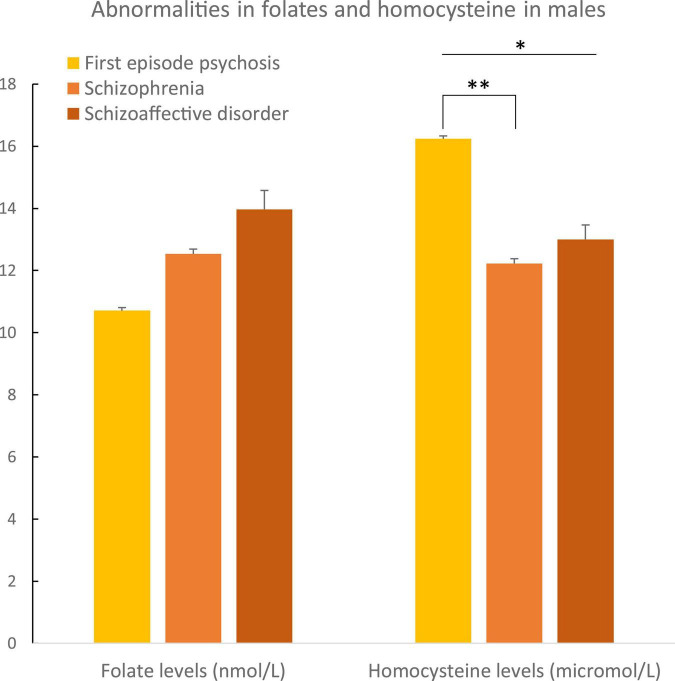
Abnormalities in folate and homocysteine in males according to psychosis spectrum diagnoses. Differences in homocysteine levels were observed between the three diagnostic groups in males (*p* = 0.04). Differences in homocysteine levels were observed between male patients with a first episode of psychosis and male patients with schizophrenia (*p* = 0.01). *p ≤ 0.05 and **p ≤ 0.01.

**FIGURE 5:**
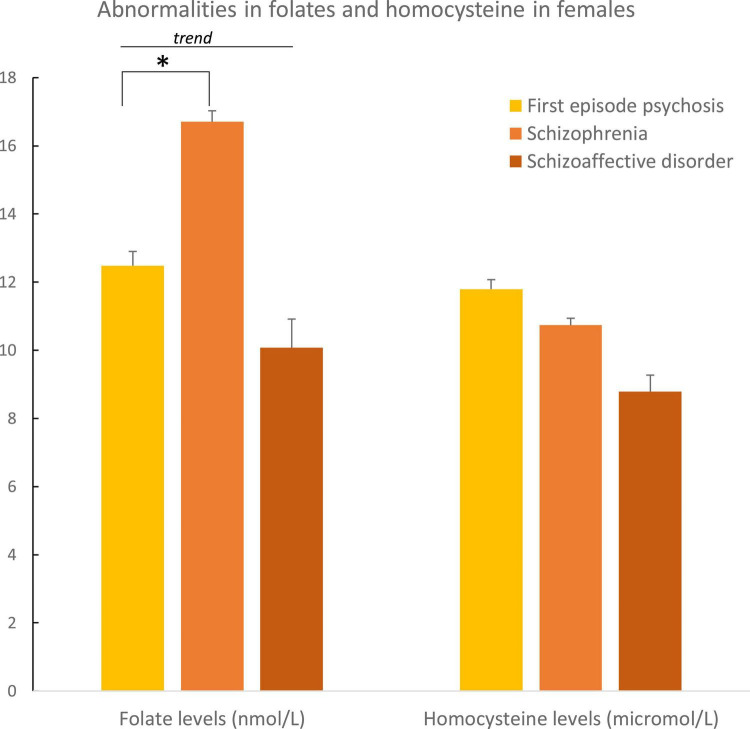
Abnormalities in folate and homocysteine in females according to psychosis spectrum diagnoses. Differences in folate levels were observed between female patients with a first episode of psychosis and female patients with schizophrenia (*p* = 0.05). *p ≤ 0.05.

In males, plasma folate levels were significantly lower (*p* = 0.02), and homocysteine higher (*p* = 0.05) than in female patients (Table 2 and Figure 3). *Post hoc* analyses on females and males separately, showed statistically significant higher hyperhomocysteinemia in FEP male patients compared to male patients with schizophrenia (*p* = 0.01, differences between the three groups had a *p* = 0.04, Table 3 and Figure 4). Analyses in female data only, showed trends for decreased levels of folate (*p* = 0.06) and folate deficiencies (*p* = 0.08) in FEP patients compared to patients with schizophrenia and schizoaffective disorders (Table 4 and Figure 5). Deficiencies in folate levels were significantly higher in FEP compared to schizophrenia female patients (*p* = 0.05).

**TABLE 3:** Biological characteristics according to diagnosis in men.

	Men (*n* = 135)	First episode psychosis (FEP; *n* = 28)	Schizophrenia (*n* = 88)	Schizoaffective disorder (*n* = 16)	*p*-value
Folate (B9), mean nmol/L (SD)	12.2 (8.2)	10.7 (5.7)	12.5 (8.7)	14.0 (9.7)	0.46
B12, mean pmol/L (SD)	307 (117)	320 (159)	310 (104)	286 (109)	0.81
Homocysteine, mean μmol/L (SD)	**13.2 (8.1)**	**16.2 (11)**	**12.2 (7.4)**	**13.0 (5.1)**	**0.04**
Folate (B9) deficiency, *N*/total (%)	31/135 (23)	7/28 (25)	18/88 (20)	4/16 (25)	0.84
Vitamin B12 deficiency, *N*/total (%)	7/133 (5)	1/28 (4)	5/88 (6)	1/16 (6)	0.90
Hyperhomocysteinemia, *N*/total (%)	23/117 (20)	6/28 (21)	10/88 (11)	4/16 (25)	0.22
One or more C1 abnormality, *N*/total (%)	47/135 (35)	11/28 (39)	25/88 (28)	8/16 (50)	0.18

Bold values represent statistically significant result (*p* ≤ 0.05).

**TABLE 4:** Biological characteristics according to diagnosis in women.

	Women (*n* = 53)	First episode psychosis (FEP; *n* = 17)	Schizophrenia (*n* = 28)	Schizoaffective disorder (*n* = 5)	*P*-value
Folate (B9), mean nmol/L (SD)	14.4 (8.2)	*12.5 (7.1)*	*16.7 (9.0)*	*13.0 (8.8)*	*0.06*
B12, mean pmol/L (SD)	314 (120)	310 (129)	323 (128)	317 (67)	0.91
Homocysteine, mean μmol/L (SD)	11.0 (4.3)	11.8 (4.5)	10.7 (4.6)	8.8 (1.9)	0.35
Folate (B9) deficiency, *N*/total (%)	*8/53 (15)*	*5/17 (29)*	*2/28 (7)*	*0/5 (0)*	*0.08*
Vitamin B12 deficiency, *N*/total (%)	1/52 (2)	1/16 (6)	0/28 (0)	0/5 (0)	0.38
Hyperhomocysteinemia, *N*/total (%)	6/45(13)	2/16 (12)	4/23 (17)	0/4 (0)	0.67
One or more C1 abnormality, *N*/total (%)	13/53 (25)	6/17 (35)	6/28 (26)	0/5 (0)	0.25

Italic values represent trend for significance (0.05 ≤ *p* ≤ 0.1).

**FIGURE 6:**
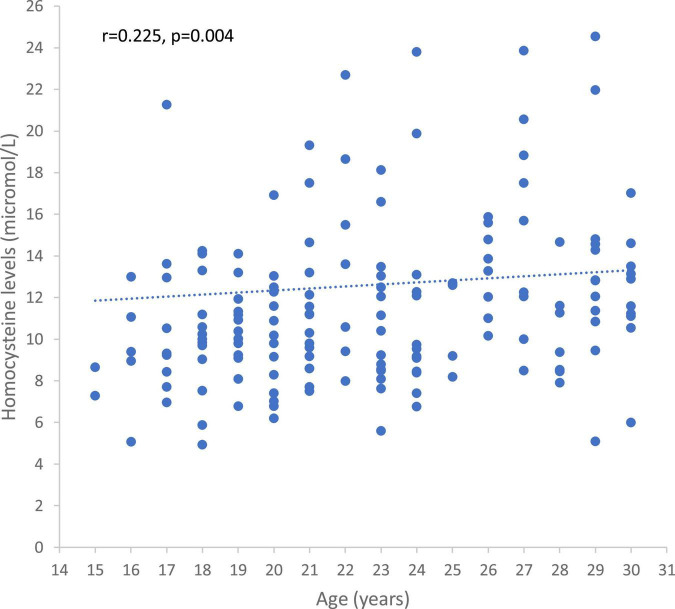
Correlation between homocysteine levels and age in the whole sample.

Homocysteinemia levels and vitamin B12 deficiency were positively correlated with age (rho = 0.225, *p* = 0.004, Figure 6 and rho = 0.172, *p* = 0.02).

#### Influence of symptoms and medication

**FIGURE 7:**
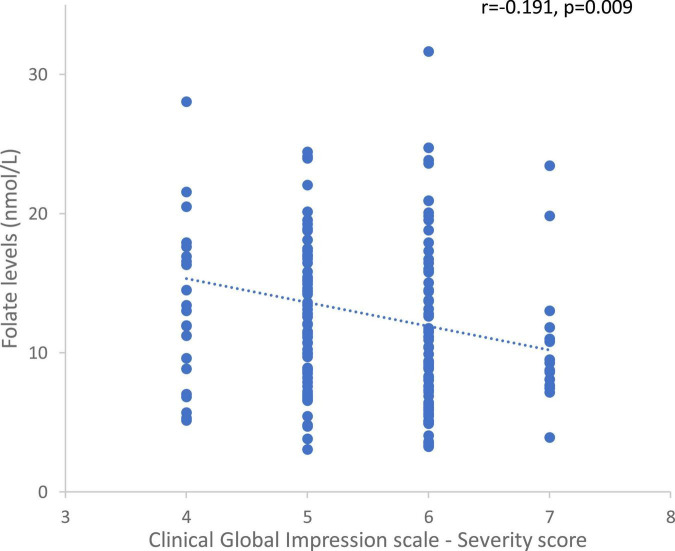
Correlation between folate levels and the Clinical Global Impression – Severity scores in the whole sample.

In the whole sample, there was an inverse correlation between plasma folate levels and the initial severity of the disorder (rho = −0.191, *p* = 0.009, Figure 7). In a *post hoc* analysis, the same correlation was found in males (rho = −0.200, *p* = 0.02), in FEP patients (rho = −0.323, *p* = 0.03), and in FEP (rho = −0.440, *p* = 0.02) and schizophrenia (rho = −0.214, *p* = 0.05) male patients.

Among the theoretical risk factors for folate deficiency, neither alcohol use disorder nor eating disorders were correlated with plasma folate levels.

No significant correlation was found between antipsychotic treatment dosage or anticonvulsant use and plasma folate levels. Conversely, a higher dosage of antipsychotic treatment at admission was associated with higher vitamin B12 levels (rho = 0.184, *p* = 0.01) and lower homocysteinemia (rho = −0.168, *p* = 0.03).

## Discussion

Our results show that a third of patients have more than one C1 abnormality, which appears to be independent of diagnostic category. The most prevalent C1 abnormality is related to folates (B9) with one fifth of patients presenting the deficiency and almost one third of FEP. Lower levels of folates were found in males compared to females and were associated with increased initial severity of the disorder in all patients.

The relatively high prevalence of folate deficiency is similar to the one found in previous studies (around 20%), which is much higher than in healthy controls (1.7 and 7%) (23, 24). Our analysis on the correlation between levels of folates and severity of psychosis also appear to show that folate deficiency, through C1 metabolism, is involved in the development and severity of schizophrenia. Indeed, we found that lower levels of folate were associated with increased initial severity of the disorder. Several studies have focused on the relationship between levels of folates and symptoms encountered in schizophrenia and FEP. Studies have shown an inverse correlation between plasma folate concentration and negative symptoms in patients with schizophrenia (23) and FEP (20, 25) and a positive correlation between negative symptoms and homocysteine levels (22, 26). Low folate levels have also been associated with the severity of negative symptoms in schizophrenia patients with specific variants in folate metabolism genes such as MTHFR or folate hydrolase 1 (FOLH1) (27). Lower folate levels in patients could arise from inadequate dietary intake (28), especially after the rapid body growth associated with adolescence. The management of negative symptoms, which affect the functional prognosis of patients, is a major therapeutic challenge and could benefit from complementary approaches such as folate supplementation (28).

Animal models of schizophrenia studies have shown protective effects of low dose folic acid on oxidative damage, cognitive impairment and immune dysfunction in pregnant and lactating rats and their offspring (29). In 1990, Godfrey et al. conducted the first therapeutic trial of L-methylfolate as an adjuvant treatment for schizophrenia. They found an increase in the rate of clinical and social remission (30). More recently, Roffman et al. reported a decrease in negative symptomatology with L-methylfolate supplementation (31). Improvement in negative symptoms was also found after 12 weeks of treatment with 2 mg/day of folate in patients with at least one MTHFR 677T risk allele (32). However, according to a more recent meta-analysis, folate supplementation effects may not be as promising as expected by original research (33). Reservations have been expressed regarding the interpretation of these therapeutic trial results, given the heterogeneity of the samples, confounding factors and the use of plasma assays. Indeed, the absence of well-defined international standards, different analysis techniques depending on the laboratory, non-matching of cases and controls on age or sex, or the failure to take into account dietary habits may prevent the relevance of meta-analyses or have consensus on the benefits of the supplementation (17). Could supplementation be helpful in the early stages of the disorder though? To the best of our knowledge, there is only one study that has supplemented patients with FEP with folates (34). This randomised controlled trial showed a beneficial effect of 12 weeks vitamin supplementation on attentional capacities compared to placebo. No effects were observed on symptoms. These neuroprotective properties are considered of importance in patients with high homocysteine levels (35), suggesting the interest in adapting the supplementation of the individual status of C1.

Based on the principles of precision medicine, treating those patients most likely to benefit is the challenge of any prescription. Among patients with early psychosis and schizophrenia, those at risk of developing folate deficiency are good candidates for supplementation. A randomised controlled trial (35) showed that genetic variations could impact the improvement in negative symptoms under folic acid and vitamin B12 treatment. These results support a personalised medical approach to the treatment of schizophrenia symptoms. Another reason to supplement only those in need of folates is that increasing dietary folic acid intake may lead to side effects. Indeed, at a dosage of more than 0.8 mg/day, serum folic acid not metabolised by dihydrofolate reductase and progressively accumulated could cross the blood-brain barrier and compete with intracerebral L-methyl folate (6), leading to a decrease in monoamine production (36) and the aggravation of some psychiatric disorders. In addition, excessive folic acid intakes could disrupt energy and lipid metabolism (37), promote tumour growth when cancer cells strongly express folate receptor alpha (38). In addition, folate supplementation could also have effects on other metabolic pathways such as oxidative stress or inflammation, which are also disrupted in the early stages of psychosis (39).

In our study, men had significantly lower plasma folate levels compared to women. A recent study found the same result, but in the control population, not in patients with chronic schizophrenia (but 59 years old) (40). Another study found that a 12-week treatment with olanzapine versus risperidone in patients with first episode schizophrenia was associated with a decrease in folate, vitamin B12 and an increase in homocysteine only in male patients who were taking olanzapine (41). It could be suggested that females have resilient mechanisms, associated with hormones or the age of onset of the disorder, which may protect them against one-carbon deficiencies observed in psychoses.

The levels of folates were inversely correlated with homocysteinemia, the second most prevalent deficiency of C1 (18%) observed in our study. The mean plasma homocysteine concentration was of 12.6 μmol/L, a relatively high level in such a young cohort of patients, if we compare it with the 9.9 μmol/L in healthy, relatively older (35–60 years), adults living in the same geographical area (42). Several studies have shown that homocysteinemia is positively correlated with age (43), a correlation that was replicated in our cohort. Our results also showed that homocysteinemia is inversely correlated with chlorpromazine equivalent dosage. The higher doses of antipsychotics the lower the levels of homocysteine, hence suggesting an indirect beneficial effect of medication via homocysteine levels. In a cohort of young male patients, homocysteinemia was elevated during decompensation and decreased during the remission phase (44), supporting this hypothesis. However, evidence of the impact of antipsychotic treatment on C1 metabolism would require pre-clinical studies and longitudinal data collection. Another puzzling result is that we found a similar association between chlorpromazine equivalents and levels of vitamin B12, but not between chlorpromazine equivalents and levels of folate. The involvement of anticonvulsant treatments in folate deficiency is known (9). No statistically significant association was found between antipsychotic treatment dosage or anticonvulsant treatment and plasma folate levels in our patient cohort. Thus, the prevalence of folate deficiency in our cohort does not appear to be associated with the treatment of these patients.

Our results should be interpreted with caution as several limitations have been identified. Firstly, we acknowledge that we would have benefited from a healthy control group, in order to compare it with the different diagnostic groups. However, this is a retrospective study and such data were unfortunately not available. In addition, we did not quantify patients’ dietary intake or explore their diet. The revision and expansion of folate threshold values by the World Health Organization (WHO) in 2008 (45) describes that folate plasma concentration of less than 10 nmol/L (4 ng/ml) or intra-erythrocyte concentration of less than 340 nmol/L (151 ng/ml) are the threshold for folate deficiency (46). However, the use of food folate supplementation in some countries and the lack of international standardisation of folate deficiency also make it difficult to compare prevalence rates of vitamin deficiency between countries. Another significant limitation is that plasma folate levels may not be a good reflection of folate status. Plasma folate is sensitive to daily variations in folate (vitamin B9) intake, unlike intra-erythrocyte folate levels, which would be a better, long-term indicator (42). However, this measure is currently the most widely used in clinical practice. Of the 145 studies in McLean’s review, 55% assessed folate status using plasma folate concentration, 21% using intra-erythrocyte concentration, and 23% using both (25). Last, plasma and intra-erythrocyte folate levels do not necessarily represent intracerebral status. The transport of folate to the central nervous system, if impaired, leads to intracerebral folate deficiency despite normal peripheral levels (47). In a small study, authors reported that 83% of their 18 patients with schizophrenia and only 3.3% of 30 controls (*p* < 0.0001) had autoantibodies to folate receptor alpha (FRα), which blocked the transfer of MTHF into the brain (6).

## Conclusion

This study supports the idea of an increased prevalence of one-carbon metabolism abnormalities, which are especially high in young patients with psychotic disorders. To our knowledge, this is the first French study to investigate these metabolic abnormalities. These results are consistent with the literature and highlight the interest of new therapeutic perspectives, such as folate supplementation. With a view to a personalised medical approach to psychotic symptoms, future studies will seek to target more precisely the population concerned by folate supplementation and the formulations (folate, folinic acid, etc.) adapted to each patient’s profile.

## Data availability statement

The datasets presented in this article are not readily available because data belong to the GHU. Requests to access the datasets should be directed to M-OK, marie-odile.krebs@inserm.fr.

## Ethics statement

Ethical review and approval was not required for the study on human participants in accordance with the local legislation and institutional requirements. Written informed consent for participation was not required for this study in accordance with the national legislation and the institutional requirements.

## Author contributions

AF and MU did the statistical analysis and wrote the manuscript under the supervision of M-OK and LS. MU conducted the data collection. FR, MP, and BC helped to improve the entire manuscript. M-OK and LS supervised the statistical analysis and the redaction of the manuscript. All authors contributed to the article and approved the submitted version.
